# A Study on Satisfaction and Influencing Factors of Educational Practice of Preschool Education Majors in Higher Vocational Colleges

**DOI:** 10.3389/fpsyg.2022.944173

**Published:** 2022-08-22

**Authors:** Jing Xiong, Heung Kou, Fang Zhang, Weifeng Kong

**Affiliations:** ^1^College of Pre-school Education, Jingzhou Institute of Technology, Jingzhou, China; ^2^College of Textile, Clothing, and Art Design, Jingzhou Institute of Technology, Jingzhou, China; ^3^College of Education, Sehan University, Yeongam-gun, South Korea; ^4^Basic Course Department, Tianjin Conservatory of Music, Tianjin, China

**Keywords:** educational practice satisfaction, influencing factors, preschool, education, student

## Abstract

In September 2020, the Ministry of Education and other nine departments issued the notice of “Action Plan for Improving the Quality of Vocational Education (2020–2023)” which emphasizes strong practical teaching. The state pays more and more attention to the quality of practical practice in higher vocational colleges. From the perspective of student satisfaction, this study explores the satisfaction of preschool education students on educational practice and related influencing factors, so as to carry out better educational practice and improve the quality of students’ educational practice in colleges and universities. This study uses the questionnaire method, according to the existing literature and theory to build the results of self-compiled “higher vocational college students majoring in preschool education practice satisfaction and influencing factors questionnaire.” A total of 463 students majoring in preschool education in higher vocational colleges were investigated. The following research results are obtained. 1. The overall satisfaction of students majoring in preschool education in higher vocational colleges to educational practice is above the average. In descending order of satisfaction question scores, other factors influencing student’s satisfaction are the tutors in the educational practice park, the educational practice base, the content of educational practice, the management of educational practice, the tutors in the school of educational practice, and the time of educational practice. 2. For constructing the influencing factor model of educational practice satisfaction, it is found that the influencing factors of educational practice satisfaction are university management rational factors, support factors from the educational base, and students’ own factors.

## Introduction

### Necessity of the Study

According to Zeng et al. (2016), with the implementation of the universal two-child policy in China, strengthening the construction of kindergartens has become an important livelihood project, Zeng et al. (2016). As higher vocational colleges are the main venue for cultivating children’s teachers, they propel important historical responsibilities.

In September 2020, the Ministry of Education and other 9 departments issued a notice on the issuance of “Action Plan for Improving the Quality and Training of Vocational Education (2020–2023)”, which emphasizes strong practical teaching. In principle, students’ internship teaching hours account for more than 50% of the total school hours. A variety of internship approaches, such as cognitive internship, follow-up internship, and post internship, can be actively implemented according to the actual professional concentration or phased arrangement.

In recent years, national policies have continuously emphasized the importance of preschool education practice in higher vocational colleges, and the demand for highly educated and high-quality kindergarten teachers has surged. Under the realistic background, it is particularly important to organize and carry out standardized educational practice, enrich the content and form of educational practice, and focus on improving students’ educational practice ability. Therefore, considering students as the main body, through the investigation of students’ satisfaction, this study can clarify the main problems existing in the current education practice, which plays an important role in the benign development of preschool education.

### Purpose of the Study

To promote the professional development of students majoring in preschool education and improve their professional ability, higher vocational colleges attach great importance to the arrangement of educational practice for college students. Liu (2018) proposed that the specific arrangements of different schools are different, but on the whole, they all include two links as follows: on-the-job internship and off-the-job internship. These links are to make students better adapt to the study of the kindergarten after moving to work.

In theory, trying to investigate the current situation of preschool educational practice satisfaction in higher vocational colleges, intuitively understanding the influencing factors of education practice satisfaction, can enrich the related theory of preschool education in higher vocational colleges.

In practice, Dan and Liu (2018) pointed out that students, as the main body of educational practice, have direct and real feelings for each practice link. Therefore, the survey of students’ satisfaction with educational practice helps to understand the problems existing in educational practice programs in a timely manner. For higher vocational colleges to better carry out education practice, colleges should improve the quality of student education practice service.

### Research Questions

This study uses the questionnaire survey method to understand the current situation of educational practice satisfaction of preschool education students in higher vocational colleges [to make this questionnaire I referred to Liu’s (2018) master’s thesis on the satisfaction and influencing factors of educational practice of preschool education students in colleges and universities], and puts forward methods and suggestions to improve the satisfaction of practice. Therefore, it is hoped that the following problems can be solved through research:

(1) What is the satisfaction of preschool education students in higher vocational colleges?

(2) Is there a gender difference in overall satisfaction?

(3) Are there any differences in overall satisfaction between different internship types (concentration and segmentation)?

(4) Are there differences in overall satisfaction among different internship locations?

(5) Is there any difference in the overall satisfaction of different internship durations?

(6) Is there any difference in overall satisfaction among different locations?

(7) Does the nature of different parks differ in overall satisfaction?

(8) Is there any difference in overall satisfaction among different garden grades?

(9) Do students’ own factors have a positive impact on students’ overall satisfaction?

(10) Do the supporting factors have a positive impact on students’ overall satisfaction?

(11) Do university management factors have a positive impact on students’ overall satisfaction?

## Theoretical Investigation

### Educational Practice

#### Definition

The definition of educational practice is divided into broad definition and narrow definition. The broad definition includes the following discussion: The encyclopedia of Chinese Education (1991) points out that educational practice is learning activity for normal college students to participate in education and teaching practice. It is an important educational link that reflects the characteristics of normal education and cultivates qualified teachers. It is an indispensable part of normal school education at all levels. Xu (2001) believes that educational practice is a comprehensive practical activity of education and teaching in normal colleges. Specifically, it is in accordance with the normal education teaching plan issued by the national education department, under the guidance of teachers, normal students actively and consciously use the acquired education theory, professional knowledge, and skills, in the internship school, directly engaged in teaching practice and ideological and moral education practice of an educational activity.

On the narrow sense of educational practice, Educational Dictionary (1998) points out that educational practice is a form of professional practice of education and teaching carried out by senior students in normal colleges and universities to practice schools, including visiting, probation, probation, acting or assisting class teachers, and participating in educational administration. The definition in Practical Education Dictionary (1995) is that Educational practice is a form of education and teaching practice for senior students in primary or secondary schools.

This study adopts the definition of educational practice in a broad sense, and believes that educational practice is a practical activity that students participate in, which is arranged by the colleges and universities to enter the practice place, and is jointly guided by the university instructors and the practice place instructors.

#### Educational Practice Mode

Educational practice mode is the process of putting educational practice plan into practice. It is the basic way to realize the goal of educational practice. Xie (2005). There are many ways to divide the types of teaching practice modes by domestic and foreign scholars. The most common way is single standard classification and comprehensive classification. According to a single standard, for example, the education practice time is divided into centralized education practice and segmented education practice. According to the location of educational practice, it is divided into fixed-point educational practice and decentralized educational practice. Kan (2011). The educational practice modes are comprehensively classified as internship decentralized mode and full-course educational practice mode. At present, Ye (2013) reported that the common educational practice modes in China include centralized fixed-point educational practice mode, “entrusted” educational practice mode, mixed formation centralized educational practice mode, directional post educational practice mode, and independent choice educational practice mode.

### Student Satisfaction

#### Definition

According to Liu (2011), the concept of students’ satisfaction came into being in the 1960’s, which is generally derived from attitude or need and expectation. Yang (2003) pointed out that students’ satisfaction is “students on learning, life and other aspects of a general emotional ideas and views.” This definition stems from attitudes. According to Li (2007), students’ satisfaction refers to a psychological feeling of joy or disappointment when students compare their actual feelings of receiving school education services with their expected expectations as customers receiving school education services. Han (2010) defined student satisfaction as “the degree of satisfaction of students, that is, the degree to which students’ actual feelings of education are compared with their expectations.”

### Student Satisfaction Model

According to Yi (2009), the American scholar Cardoco first introduced the concept of “customer satisfaction” into sales field in 1965. Since then scholars have paid attention to customer satisfaction and introduced this concept into other fields. The concept of customer satisfaction was introduced in China in the late 1990’s. The United States is the first country to use college students’ satisfaction in the quality evaluation of higher education (Liang, 2007). China began to carry out research on college students’ satisfaction in the early 21st century (Li, 2016).

The United States was the first to apply the satisfaction measurement to the measurement of service quality in colleges and universities. In 1966, the United States used the CIRP to measure the satisfaction of freshmen. In 1994, the SSI scale was used to measure the satisfaction of college students across the United States.

The Swedish SCSB model is the earliest national customer satisfaction index model in the world, which is composed of five latent variables as follows: customer expectations, value perception, customer satisfaction, customer complaints, and customer loyalty. The ACSI model of the United States is the most widely used and used customer satisfaction index model, which is composed of six latent variables as follows: customer expectation, quality perception, value perception, customer satisfaction, customer complaints, and customer loyalty. Based on the ACSI model, China constructs the CCSI model, including 6 latent variables as follows: brand image, expected quality, quality perception, value perception, user satisfaction, and user loyalty. Till present, there is no unified higher education student satisfaction index model in China. Most of them look for factors that affect student satisfaction and construct structural equation models on the basis of referring to foreign customer satisfaction index models (Liu, 2011).

### Preschool Education Majors in Higher Vocational Colleges

#### Higher Vocational Colleges

“Higher Vocational and Technical School” is referred to as higher vocational colleges, which includes two educational levels of college and undergraduate education (Li, 2016). In terms of training objectives, higher vocational colleges focus on the cultivation of practical, technical, and skilled high-tech applied talents for grassroots production, service, and management, and on the cultivation of students’ practical application ability. In terms of school system, the school system in higher vocational colleges is generally 3 years. The higher vocational colleges referred to in this study are mainly 3-year colleges (Cheng, 2020).

#### Preschool Education

Preschool education in the Education Dictionary specifically refers to the education of preschool children, and “specialty” refers to a department or secondary specialized school in colleges and universities, which classifies academic studies according to the scientific division of labor or the division of labor in the production sector. Therefore, preschool education specialty refers to the professional categories generated by education for preschool children in institutions of higher learning or secondary professional schools. Preschool education specialty in this study refers to preschool education specialty in higher vocational colleges (Gu, 2002).

With regard to the training objectives of preschool education major in higher vocational colleges, the “Education Dictionary” proposes that it cultivates students with preschool education-related professional knowledge, good professional ethics, and professional ability, and can be competent for various preschool education-related studies after graduation, such as front-line teachers in preschool education institutions, care givers, and preschool education-related management personnel. Li (2016) believed that the students should have high moral character, profound culture, superb skills, correct political concepts and moral concepts and advanced preschool education concepts, solid teaching ability, solid “talking, doing, jumping” skills, good communication, and innovative grassroots preschool educators. Cheng (2020) believed that preschool education major in higher vocational colleges is located in preschool education-related enterprises and institutions, such as preschool education institutions, early childhood education institutions, and preschool education-related training institutions. It aims to cultivate applied talents with relevant knowledge, professional concepts, professional ethics, and professional ability of preschool education major, and focuses on basic application in education, reflecting “normal” and “application.”

### Preschool Education Students’ Satisfaction With Educational Practice

#### Definition

Liu (2018) believes that preschool education students’ satisfaction refers to the psychological feelings of preschool education students after participating in educational practice and comparing their actual gains and expectations in practice, which mainly displays in the education practice time, the education practice content, the education practice base, the education practice management, the education practice school instructor, and the education practice park instructor aspect satisfaction.

#### Student Satisfaction Model of Educational Practice

The survey content of educational practice satisfaction of each major is different, depending on the particularity of each major. Wu (2012) conducted a self-designed questionnaire survey on the satisfaction of medical students in clinical practice, including the investigation of hospital teaching conditions, teachers, practice units, practice management, and practice effect. Xu (2013) investigated the satisfaction of hotel interns in higher vocational colleges from five aspects as follows: internship work itself, internship working environment, internship allowance, personal development, and interpersonal relationship. Zhou (2016) investigated the current situation of satisfaction with physical education practice in normal universities from four aspects as follows: students’ own ability, students’ colleges, instructors, and practice bases. Hao (2017) investigated the satisfaction of students majoring in physical education in colleges and universities from the aspects of time, mode, school, teacher, and practice content.

Zeng et al. (2016) investigated the satisfaction of preschool education students in educational practice from the aspects of practice time, practice cognition, practice behavior, teacher guidance and salary. Liu (2018) investigated the satisfaction degree of preschool education professional education practice, including the time, content, practice base, kindergarten teachers, practice management, and college teachers.

### Literature Review of Preschool Education Students’ Satisfaction With Teaching Practice

Based on the above literature, it is found that there are many studies on the satisfaction of preschool education students’ educational practice at home and abroad. Relevant researchers from a unique research perspective based on a large number of reliable data, through quantitative or qualitative methods, explore the influencing factors, structure, and relationship of preschool education students’ educational practice satisfaction, and achieve certain results. However, there are some shortcomings in previous studies. First of all, from the perspective of research objects, most studies are about undergraduate students. However, there are a few studies on college students in higher vocational colleges. It was believed that the research perspective has great value; second, most scholars in the study of preschool education major in higher vocational college education practice satisfaction present situation, only satisfaction present situation analysis, and then according to the preschool education major students’ education practice satisfaction present situation put forward the corresponding coping strategies. However, the satisfaction of students’ educational practice is the subjective feeling of all aspects before and after their internship. It is also of great significance to study the influencing factors of students’ educational practice satisfaction.

Therefore, this study aims to make up for the lack of previous research literature, considering preschool education students in higher vocational colleges as the research object, analyze the current situation of preschool education students’ satisfaction with educational practice in higher vocational colleges, and explore the influencing factors of preschool education students’ satisfaction in higher vocational colleges. It is hoped that through the discussion of these problems, effective suggestions can be put forward to improve students’ internship satisfaction and improve the quality of preschool education in higher vocational colleges. This study has a clear research object, a clear division of disciplines, a wide range of sample sources, and a reasonable distribution. Therefore, the conclusion is scientific, rigorous, more convincing, and credible, and it is believed that it can promote existing research.

## Research Design

### Research Object

According to the research purpose, the preschool education students in higher vocational colleges in Jingzhou City, Hubei Province, China are selected as the research objects by random sampling. A total of 463 questionnaires were distributed, and 424 valid studies were recovered, with an effective recovery rate of 91 % (see Table 1).

**TABLE 1:** General features of the participants (Total *N* = 424).

Variable	Type	*N*	Percentage (%)
Gender	Male	41	9.7
	Female	383	90.3
Practice form	Centralized practice	331	78.1
	Segmented practice	93	21.9
Number of internships	1 s	35	8.3
	2 s	287	67.7
	3 s	59	13.9
	4 More than times	43	10.1
Internship duration	4 Weeks and below	152	35.8
	5 ∼ 8 weeks	17	4
	9 ∼ 12 weeks	14	3.3
	13 ∼ 16 weeks	21	5
	16 More than weeks	220	51.9
Practice type	Fixed point practice	329	77.6
	Independent practice	95	22.4
Park location	City	280	66
	Countryside	81	19.1
	Blend	63	14.9
Nature of park	Public	112	26.4
	Civilian-run	216	50.9
	Blend	96	22.6
Kindergarten grade	Demonstration Park	99	23.3
	Class I	47	11.1
	Class II	56	13.2
	Three classes of initials	17	4
	No grade	11	2.6
	Hear nothing of	194	45.8
Number of school instructors	1–5 people	139	32.8
	6–10 people	67	15.8
	11–15 people	38	9
	15 More than people	180	42.5
Class during internship	Toban	14	3.3
	A reception class	96	22.6
	Middle shift	116	27.4
	Big class	106	25
	Rotation	92	21.7

### Research Process

#### Questionnaire on the Satisfaction of Preschool Education Majors in Higher Vocational Colleges

In this study, through the combing and analysis of relevant literature, drawing on the structure and content of Liu’s questionnaire, referring to the manual of college education practice plan and through expert evaluation, the “Questionnaire on the Satisfaction of Preschool Education Majors in Higher Vocational Colleges” was designed. In Liu’s questionnaire, the reliability coefficient of educational practice satisfaction of students majoring in preschool education in Colleges and Universities is as follows: the Cronbach’s alpha value for satisfaction of tutors from kindergartens is 0.965, for the satisfaction of tutors within the college is 0.949, for the satisfaction of educational practice contents is 0.908, for the satisfaction of education practice management is 0.870, for the satisfaction of education practice base is 0.904, and for the satisfaction of education practice time span is 0.844, and the Cronbach’s alpha value for overall satisfaction is 0.955, all of which are above 0.8, indicating that the questionnaire bears good reliability and stability.

The first part of my questionnaire is the basic information of students and the basic situation of participating in educational practice, including the school, gender, educational practice time, the number of educational practice, educational practice base, the nature of the educational practice park, the level of the educational practice park, the class of the educational practice base, the instructor of the educational practice park, the preparation before the practice, and the evaluation of the practice. Topic types include filling in the blank, single choice, and multiple choice. The second part is the questionnaire of students’ satisfaction with teaching practice of preschool education major in higher vocational colleges, including 24 questions and 6 dimensions, including teaching practice time, teaching practice content, teaching practice base, teaching practice management, teaching practice school teachers, and teaching practice park teachers. The questionnaire adopts five-level Likert rating form.

#### Questionnaire on the Influencing Factors of Satisfaction of Preschool Education Majors in Higher Vocational Colleges

Based on the review and analysis of relevant literature, this study draws on the structure and content of Liu’s questionnaire, and draws on some items in the American ACSI model and Chinese CCSI model. In Liu’s questionnaire, for all the influencing factors of educational practice for preschool education majors in Colleges and Universities, the Cronbach’s α value on Kindergarten support factor is 0.959, on students’ own factor is 0.925, and on university management factor is 0.915, which are all above 0.8, indicating that the questionnaire has good reliability and stability. The structure and content of the questionnaire on the influencing factors of preschool education students’ satisfaction with educational practice in higher vocational colleges are obtained. There are a total of 34 topics, in three dimensions, namely students’ own factors, university management factors, and garden support factors. The questionnaire adopts five-level Likert rating form.

#### Analytical Methods

The SPSS 24.0 software was used to input and process the data. On the basis of descriptive statistics on the satisfaction of preschool education students in higher vocational colleges, correlation analysis and regression analysis are carried out on the collected sample data.

## Analysis Results

### Reliability Test

#### Reliability Test of the Educational Practice Satisfaction Questionnaire

From Table 2, it can be seen that the Cronbach’s α value for all topics is 0.975, and the Cronbach’s α values for the following topics are as follows: the Cronbach’s α value for educational internship time span is 0.897, for educational practice content is 0.914, for educational practice base is 0.946, for educational practice management is 0.842, for tutors from college is 0.908, and for tutors from educational practice base (e.g., kindergarten) is 0.953, indicating that the overall reliability of questionnaire and the sub dimensional reliability are excellent. It shows that the collected data have relatively good internal consistency, and the questionnaire has good stability and reliability.

**TABLE 2:** Reliability coefficients of educational practice satisfaction of preschool education majors in colleges and universities.

Variables	Cronbach’s α	Number of topics
Overall satisfaction	0.975	26
Educational practice time	0.897	3
Content of educational practice	0.914	6
Educational practice base	0.842	3
Educational practice management	0.954	4
Educational practice school instructor	0.908	5
Instructor of education practice Park	0.953	5

#### Reliability Test of the Questionnaire on the Influencing Factors of Educational Practice

From Table 3, it can be seen that the Cronbach’s α value for all influencing factors is 0.980, for students’ own factors is 0.944, for school management factors is 0.963, and for supporting factors of the park is 0.951. The results indicate that the reliability of the total questionnaire and of the sub dimensions is excellent. It shows that the collected data have relatively good internal consistency, and the questionnaire has good stability and reliability.

**TABLE 3:** Reliability coefficient of the influencing factors of educational practice on preschool education majors in colleges and universities.

Variables	Cronbach’s α	Number of topics
Overall influencing factors	0.980	34
Students’ own factors	0.944	10
School management factors	0.963	14
Supporting factors of the park	0.951	10

### Descriptive Statistics on the Satisfaction of Preschool Education Majors in Higher Vocational Colleges

To understand the level of the main variables in this study, descriptive statistical analysis is carried out. The average value, standard deviation, skewness, and kurtosis of the comparison are shown. The average calculation results are as follows: the average value of overall satisfaction is 3.92, and the standard deviation is 0.61, which is at the middle-upper level. The average value of students’ own factors is 3.93, and the standard deviation is 0.62, which is at the middle-upper level. The average value of garden support factors is 3.91, and the standard deviation is 0.69, which is at the middle-upper level. The average value of school management factors is 3.91, and the standard deviation is 0.68, which is at the middle-upper level. By using skewness and kurtosis identification, it showed less than 3, which confirmed that the data were approximate normal distribution. In addition, we made a descriptive statistical analysis of the 6 sub-dimensions of overall satisfaction. The average time of educational practice is 3.78, and the standard deviation is 0.77, which is above the middle level. The average content of educational practice is 3.95, and the standard deviation is 0.63, which is above the middle level. The average value of educational practice base is 3.97, and the standard deviation is 0.69, which is above the middle level. The average value of educational practice management is 3.94, and the standard deviation is 0.71, which is above the middle level. The average value of school instructors in educational practice is 3.80, and the standard deviation is 0.70, which is above the middle level. The average value of the teachers in the education practice park is 3.99, and the standard deviation is 0.69. Similarly, the skewness and kurtosis showed less than 3, which confirmed that the data were approximately normal distribution (see Table 4).

**TABLE 4:** Descriptive statistical analysis results of each dimension.

Dimension	*N*	Min	Max	*M*	*SD*	Skewness	Kurtosis
Overall satisfaction	424	1.85	5.00	3.92	0.61	−0.20	0.15
Students’ own factors	424	2.00	5.00	3.93	0.62	−0.21	−0.03
Supporting factors of the park	424	1.14	5.00	3.91	0.69	−0.54	0.79
School management factors	424	1.10	5.00	3.91	0.68	−0.38	0.42
Educational practice time	424	1.00	5.00	3.78	0.77	−0.30	0.25
Content of educational practice	424	2.00	5.00	3.95	0.63	−0.12	−0.28
Educational practice base	424	1.00	5.00	3.97	0.69	−0.46	0.69
Educational practice management	424	1.00	5.00	3.94	0.71	−0.58	0.87
Educational practice school instructor	424	1.25	5.00	3.80	0.70	−0.32	0.43
Instructor of education practice Park	424	1.00	5.00	3.99	0.69	−0.59	0.98

### Correlation Analysis of Preschool Education Students’ Educational Practice Satisfaction in Higher Vocational Colleges

**FIGURE 1:**
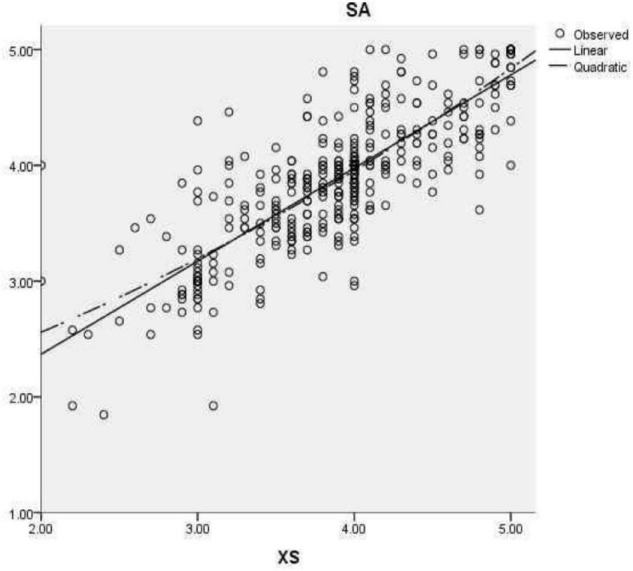
Linear and curve fitting diagram of students’ own factors (XS) to overall satisfaction (SA).

**FIGURE 2:**
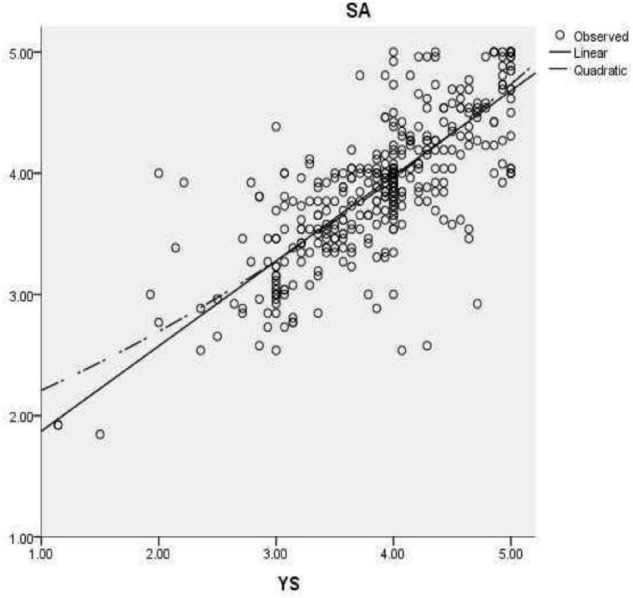
Linear and curve fitting diagram of kindergarten supported factors (YS) to overall satisfaction (SA).

**FIGURE 3:**
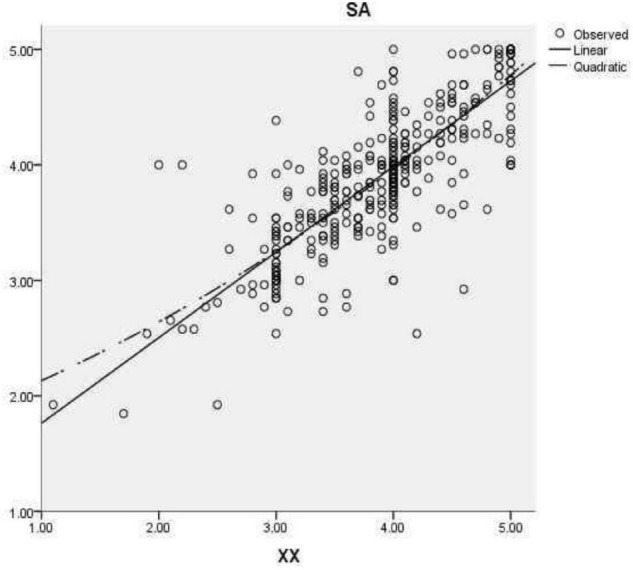
Linear and curve fitting diagram of school management factors (XX) to overall satisfaction (SA).

To determine the correlation between overall satisfaction, students’ own factors (see Figure 1), kindergarten support factors (see Figure 2), and school management factors (see Figure 3), correlation analysis was carried out. As shown, there was a significant positive correlation between students’ overall satisfaction and students’ own factors (*r* = 0.823), kindergarten support factors (*r* = 0.798), and school management factors (*r* = 0.823). There was a significant positive correlation between the students’ own factors and the factors supported by the kindergarten (*r* = 0.789), and between the students’ own factors and the school management factors (*r* = 0.814). There was a significantly positive correlation between the garden support factors and school management factors (*r* = 0.864) (see Table 5).

**TABLE 5:** Correlation analysis results of each dimension.

	OS	SOF	SFP	SMF
OS	1			
SOF	0.823**	1		
SFP	0.798**	0.789**	1	
SMF	0.823**	0.814**	0.864**	1

**p < 0.01.

*OS, Overall satisfaction; SOF, Students’ own factors; SFP, Supporting factors of the park; SMF, School management factors.*

### The Regression Analysis of Preschool Education Students’ Educational Practice Satisfaction in Higher Vocational Colleges

To test the influence of students’ own factors, kindergarten support factors, and school management factors on overall satisfaction, multiple regression analyses were carried out. The overall level results are shown. The descriptive power of the regression model is about 75.7 %, and the model is significant as a whole (*F* = 435.221, *p* < 0.001). Students’ own factors (β = 0.403, *t* = 9.281, *p* < 0.001), park support factors (β = 0.208, *t* = 4.150, *p* < 0.001), and school management factors (β = 0.315, *t* = 5.955, *p* < 0.001) can all significantly positively affect overall satisfaction (see Table 6).

**TABLE 6:** Multiple linear regression analysis results of overall satisfaction on students’ own factors, kindergarten support factors, and school management factors.

Model		Non-standardized coefficient		Standardization coefficient	t	Significance	Collinearity statistics	
		B	Standard error	Beta			Tolerance	VIF
1	Constant	0.548	0.095		5.751	0.000		
	Students’ own factors	0.393	0.042	0.403	9.281	0.000	0.308	3.248
	Supporting factors of the park	0.183	0.044	0.208	4.150	0.000	0.231	4.323
	School management factors	0.284	0.048	0.315	5.955	0.000	0.207	4.836

To test the fitting results of students’ own factors, garden support factors, and school management factors on overall satisfaction, linear and curve fitting were carried out. The results are shown as follows. In the linear equation, the students’ own factors can explain 67.8 % of the variation of overall satisfaction, and the model is generally indigenous (*F* = 888.059, *p* < 0.001). The fitting equation is overall satisfaction = 0.805 × students’ own factors+0.759. In the curve equation, the students’ own factors can explain 68.1 % of the variation of the overall satisfaction, and the model is significant (*F* = 449.112, *p* < 0.001). The fitting equation is overall satisfaction = 0.062 × students’ own factors 2+0.325 × students’ own factors+1.660; the results suggest that both fitting methods can reflect the impact of students’ own factors on overall satisfaction.

Similarly, in the linear equation, the garden support factor can explain 63.7 % of the variance of overall satisfaction, and the model is obvious (*F* = 740.008, p < 0.001). The fitting equation is overall satisfaction = 0.702 × garden support factor+1.170; in the curve equation, the garden support factor can explain 64.0 % of the variance of overall satisfaction, and the model is obvious (*F* = 374.996, *p* < 0.001). The fitting equation is overall satisfaction = 0.049 × garden support factor 2+0.339 garden support factor+1.822; the results suggest that the two fitting methods can reflect the impact of garden support factors on overall satisfaction.

In the linear equation, the school management factors can explain 67.7 % of the variance of overall satisfaction, and the model is obvious (*F* = 883.553, *p* < 0.001). The fitting equation is overall satisfaction = 0.741 × school management factors+1.022. In the curve equation, school management factors can explain 68.0% of the variance of overall satisfaction, and the model is obvious (*F* = 447.448, *p* < 0.001). The fitting equation is overall satisfaction = 0.051 × school management factors 2+0.356 × school management factors+1.725; these results suggest that the two fitting methods can reflect the impact of school management factors on overall satisfaction (see Table 7).

**TABLE 7:** Fitting results of Students’ own factors, supporting factors of the park, and school management factors on overall satisfaction.

Predictive variable	Equation	R^2^	Adj.R^2^	F	Constant	b1	b2
Students’ own factors	linear	0.678	0.677	888.059***	0.759	0.805	
	curve	0.681	0.679	449.112***	1.660	0.325	0.062
Supporting factors of the park	linear	0.637	0.636	740.008***	1.170	0.702	
	curve	0.640	0.639	374.996***	1.822	0.339	0.049
School management factors	linear	0.677	0.676	883.553***	1.022	0.741	
	curve	0.680	0.679	447.448***	1.725	0.356	0.051

***p < 0.001.

### Difference Analysis of Preschool Education Students’ Educational Practice Satisfaction in Higher Vocational Colleges

It can be seen from Table 8 that the difference in overall satisfaction of different internship forms is not statistically significant (*t* = –0.481, *p* = 0.631 > 0.05). Different types of internships in the overall satisfaction of the difference were statistically significant (*t* = –2.990, *p* = 0.003 < 0.01), the results suggest that fixed-point internship in the overall satisfaction score was significantly lower than that of the independent internship in the overall satisfaction score. The differences in overall satisfaction with different internships were statistically significant (*F* = 8.557, *p* < 0.001). Multiple comparison results suggested that the scores of overall satisfaction with more than four internships were significantly higher than those with one, two, and three internships. The total satisfaction score of 3 internships was significantly higher than that of 1 internship.

**TABLE 8:** Difference in test results of demographic variables in each dimension.

Demographic variables	Category	Mean ± standard deviation	t/F	p	LSD
Practice form	Centralized practice (*n* = 331)	3.91 ± 0.63	−0.481	0.631	
	Segmented practice (*n* = 93)	3.94 ± 0.55			
Practice type	Fixed point practice (*n* = 329)	3.87 ± 0.61	−2.990	0.003	
	Independent practice (*n* = 95)	4.08 ± 0.57			
Number of internships	1 (*N* = 35)	3.74 ± 0.73	8.557	<0.001	4 > 3,2,1; 3 > 1;
	2 (*N* = 287)	3.86 ± 0.57			
	3 (*N* = 59)	4.00 ± 0.62			
	4 More than times (*n* = 43)	4.31 ± 0.57			
Internship duration	4 Weeks and below (*n* = 152)	3.94 ± 0.50	2.139	0.075	
	5 ∼ 8 weeks (*n* = 17)	4.18 ± 0.50			
	9 ∼ 12 weeks (*n* = 14)	3.75 ± 0.66			
	13 ∼ 16 weeks (*n* = 21)	4.14 ± 0.59			
	16 More than weeks (*n* = 220)	3.87 ± 0.68			
Park Location	City (*n* = 280)	3.95 ± 0.54	3.290	0.038	1, 3 > 2;
	Rural (*n* = 81)	3.76 ± 0.74			
	Mixing (*n* = 63)	3.97 ± 0.70			
Nature of Park	Public (*n* = 112)	4.08 ± 0.55	6.180	0.002	1 > 2;
	Private (*n* = 216)	3.83 ± 0.59			
	Mixing (*n* = 96)	3.93 ± 0.69			
Kindergarten grade	Demonstration Park (*n* = 99)	4.05 ± 0.59	6.015	< 0.001	1, 3 > 5, 6;2, 4, 6 > 5;
	Class I (*n* = 47)	3.88 ± 0.61			
	Class II (*n* = 56)	4.07 ± 0.47			
	Class III (*n* = 17)	4.08 ± 0.48			
	No grade (*n* = 11)	3.19 ± 0.64			
	Don’t know (*n* = 194)	3.84 ± 0.63			
	6–10 Person (*n* = 67)	3.88 ± 0.57			
	11–15 Person (*n* = 38)	3.96 ± 0.53			
	15 More than (*n* = 180)	3.90 ± 0.66			

There was no significant difference in the overall satisfaction of different internship durations (*F* = 2.139, *p* = 0.075 > 0.05). The difference in overall satisfaction between different park locations was statistically significant (*F* = 3.290, *p* = 0.038 < 0.05). Multiple comparison results suggested that the scores of urban and mixed park locations in overall satisfaction were significantly higher than those of rural parks. The difference in the overall satisfaction of different park natures was statistically significant (*F* = 6.180, *p* = 0.002 < 0.01). Multiple comparison results suggested that the score of overall satisfaction of public park nature was significantly higher than that of private park nature. The difference in overall satisfaction among different grades of kindergartens was statistically significant (*F* = 6.015, *p* ≤ 0.001). The results of multiple comparison showed that the scores of overall satisfaction in the first and second grades of kindergartens were significantly higher than those in the non-grade and unknown grades of kindergartens. The scores of overall satisfaction with the first class, the third class, and the ignorance of the park level are significantly higher than those of overall satisfaction without the park level.

## Conclusion and Recommendations

### Conclusion

#### The *Status Quo* of Educational Practice Satisfaction of Preschool Education Students in Higher Vocational Colleges

(i). Students’ overall satisfaction with teaching practice is above average

We found that for students majoring in preschool education in the higher vocational college we investigated, their overall satisfaction and satisfaction in each influencing factors are between 3 and 4. Compared with the average theoretical value of 3, it shows that their satisfaction in overall and each specific factors are above average. The students’ satisfactions in descending order are: tutors from kindergartens, education practice base, educational practice contents, education practice management, tutors within the higher vocational college, and education practice time span.

(ii). There are significant differences in the demographic variables of satisfaction with educational practice

In this study, from the form of educational practice, practice type, number of educational practice, practice time, place of educational practice, the nature of educational practice park, the level of educational practice park, 7 aspects of higher vocational college preschool education students’ satisfaction with educational practice in 6 dimensions were tested. The results show that the students’ satisfaction of independent education practice is higher than that of fixed-point education practice, the students’ satisfaction of urban education practice is higher than that of rural education practice, the students’ satisfaction of public education practice is higher than that of private education practice, and the students’ satisfaction of education practice in the first and second class gardens is higher than that of other gardens (see Table 8).

#### Influencing Factors of Educational Practice Satisfaction of College Students Majoring in Preschool Education

Based on the research results of customer satisfaction evaluation and college students’ satisfaction evaluation at home and abroad, especially the American customer satisfaction index model (ACSI) and Chinese customer satisfaction index model (CCSI), this study introduces the concept of customer satisfaction into the evaluation of educational practice in higher vocational colleges.

Based on the premise that educational practice is an educational service provided by colleges and universities and practice parks, this study combines the particularity of educational practice of preschool education major, and summarizes the factors and indicators that affect the satisfaction of educational practice of preschool education major students in higher vocational colleges. Finally, the factors that affect the satisfaction of educational practice of preschool education major students in higher vocational colleges include management factors of higher vocational colleges, support factors of kindergartens, and students’ own factors.

### Recommendations

#### Selecting High-Quality Internship Parks to Build a Dynamic Evaluation and Screening Mechanism

The survey results of this study show that students’ satisfaction with educational practice in cities, public parks, and demonstration parks where internship parks are located is high. Some rural kindergartens, private kindergartens, and ungraded kindergartens have some problems to some extent, such as backward educational concepts, outdated educational equipment, weak teachers, and relatively backward educational environment, which may affect students’ satisfaction and quality of educational practice. Therefore, higher vocational colleges should select high-quality internship parks for students. These parks should understand and accept the concept of educational practice in higher vocational colleges, and put it into practice to provide students with a variety of practical opportunities.

Higher vocational colleges should improve the selection criteria, establish professional assessment groups, and determine the indexes of the internship park, such as the location, nature and grade of the park, and select more urban kindergartens, public kindergartens, and kindergartens with higher grades. After the selection of practice parks, higher vocational colleges should regularly return to the practice parks, listen to the feedback of students, replace the unqualified practice parks in time, increase the standard practice parks, and form a dynamic evaluation and screening mechanism.

#### Carrying Out Regular Evaluation of Students’ Satisfaction With Educational Practice to Highlight Students’ Dominant Position

Students’ satisfaction with educational practice is one of the important bases for evaluating the quality of educational practice. In educational practice, students, as the main body of practice, have the most direct experience of the whole process of educational practice. Considering students’ satisfaction with educational practice as one of the criteria for evaluating the quality of educational practice in higher vocational colleges is of great significance to improve the educational practice scheme.

The results of this study show that students’ satisfaction with educational practice management is above average. Higher vocational colleges should regularly carry out students’ satisfaction evaluation of educational practice, timely find out the problems existing in educational practice, actively improve educational practice programs, and improve the quality of educational practice. The assessment can be conducted through questionnaires and individual interviews. For example, after the end of educational practice, higher vocational colleges can carry out a questionnaire survey on students’ satisfaction with educational practice in a timely manner, investigate students’ satisfaction with internship harvest, garden support, internship management, and other aspects, and allow internship instructors to select some students for individual interviews to understand students’ real ideas about educational practice, so as to continuously improve educational practice programs in higher vocational colleges.

## Data Availability Statement

The raw data supporting the conclusions of this article will be made available by the authors, without undue reservation.

## Author Contributions

All authors listed have made a substantial, direct, and intellectual contribution to the work, and approved it for publication.
